# Non-targeted metabolomics reveals the taste variations during *Baccaurea ramiflora* Lour. fruit maturation

**DOI:** 10.3389/fpls.2024.1420231

**Published:** 2024-07-08

**Authors:** Chongcheng Yang, Jiaqi Chen, Yang Zhang, Jianjian Huang, Huachen Wang, Jie Chen

**Affiliations:** ^1^ College of Coastal Agricultural Sciences, Guangdong Ocean University, Zhanjiang, China; ^2^ Organic Biology Group, Jiangxi Ganzhou Eco-environmental Monitoring Center, Ganzhou, China; ^3^ School of Food Engineering and Biotechnology, Hanshan Normal University, Chaozhou, China

**Keywords:** *Baccaurea ramiflora*, fruit flavor, taste biomarker, metabolites profiling, non-targeted metabonomics

## Abstract

*Baccaurea ramiflora* Lour. is a new kind of underutilized wild fruit tree; the metabolic reasons for its fruit flavor changes are not yet clear. In this study, the pink flesh of this excellent tasting fruit (BR) was used to reveal the metabolic causes of taste variations through five developmental stages. We identified 154 common differential metabolites of different developmental stages based on non-targeted metabolomics analysis. The accumulation of sugar and fatty acids increased significantly after 73 days, while citric acid decreased significantly. Flesh color accumulation mainly occurred 53 days ago, and vitamin accumulation occurred after 93 days. Interestingly, L-sorbose and 5-hydroxyindole-3-acetic acid were positively correlated with the sugar–acid ratio but negatively correlated with titratable acids. It indicated that L-sorbose and 5-hydroxyindole-3-acetic acid may be taste biomarkers of BR *B. ramiflora*. The results provided new metabolic lines of evidence for the taste variation during the ripening process of *B. ramiflora*.

## 1 Introduction

*Baccaurea ramiflora* Lour. (Phyllanthaceae family) is a wild fruit tree native to tropical and subtropical regions, widely distributed in South China (Hainan, Lianjiang Guangdong and South Guangxi) and Southern Yunnan, as well as in South and Southeast Asian countries such as India, Nepal, Myanmar, the Andaman Islands, Thailand, and Peninsular Malaysia (Ferrer et al., 2008; Huang et al., 2021). *B. ramiflora* is a new type of underutilized wild fruit tree with good edible quality, which is suitable for fresh consumption or processed into fruit juice, dried fruit, jam, and wine (Nesa et al., 2018). Generally, *B. ramiflora* has a high economic value potential in which the yield of an adult plant reaches 100–150 kg (Pandey et al., 2018). However, *B. ramiflora* has not yet been commercially planted and utilized, although local residents usually collect these fruits from the forests and sell them in the local market (Banerjee et al., 2022).

*B. ramiflora* has a highly edible flesh with a rich dietary fiber; it approximately contains 35.6% water, 51.9% carbohydrate, 5.58% protein, and 20.4% fiber (Ramjan and Raghavan, 2018). The fruit is rich in magnesium (504 mg/100 g), potassium (730 mg/100 g), phosphorus (132 mg/100 g), and iron (100 mg/100 g) (Ramjan and Raghavan, 2018). In addition, *B. ramiflora* has good health and medicinal—anti-inflammatory, antioxidant (Usha et al., 2014), hypoglycemic, and hypolipidemic (Ullah et al., 2012)—properties. Despite being a wild fruit, *B. ramiflora* has gradually attracted widespread attention because of its unique advantages, namely, good taste, high nutrition value, and therapeutic applications (Huang et al., 2024). Although the various local strains of *B. ramiflora* have not been well developed and utilized, BR (white pericarp and pink flesh) in Fangchenggang, Guangxi is a more popular fruit with excellent taste.

The fruit taste of *B. ramiflora* is similar to jujuba (Guo et al., 2015), apple (Ma et al., 2015), and wampee (Yin et al., 2022), ranging from sour, sweet, to astringent mainly depending on the growth stage. Primary metabolites, particularly carbohydrates and organic acids, are closely related to the sweet and sour taste of the fruit (Jiang et al., 2023). Metabolomics was increasingly used to study fruit flavors (Liu et al., 2024; Su et al., 2024). Deng et al. studied the key substances determining the flavor of rambutan fruits using widely targeted metabolomics analysis and examined the variations of the major metabolites, such as sugars, organic acids, and amino acids, throughout the maturation process of rambutan fruits (Deng et al., 2023). Chen et al. studied the key substances determining the flavor of the *B. ramiflora* fruit by non-targeted metabolomics and explored the quantities and main types of major metabolites such as sugar, organic acid, and amino acid (Chen et al., 2023). It is very important to understand the metabolic pathway and key metabolites of fruit ripening in different stages to improve the quality of *B. ramiflora.* However, the important taste-related metabolites and the underlying mechanisms, which change during maturation, are not known and have not been characterized. Limited data constrain the systematic analysis of primary and secondary metabolites that affect fruit taste.

In this study, to better understand the flavor changes of *B. ramiflora* pulp, various metabolites, such as carbohydrates, organic acids, amino acids, fatty acids, and flavonoids at different mature stages of *B. ramiflora*, were analyzed by liquid chromatography with tandem mass spectrometry (LC-MS/MS; a fast and reliable method for the detection of various plant metabolites) (Yue et al., 2018). Through the non-targeted metabolomics analysis of five typical growth stages of the BR *B. ramiflora* fruit, all metabolites were screened out. Finally, pathway enrichment analysis identified its characteristic metabolites and potential flavor biomarkers and explained their possible mechanisms.

## 2 Materials and methods

### 2.1 Plant materials

**Figure 1:**
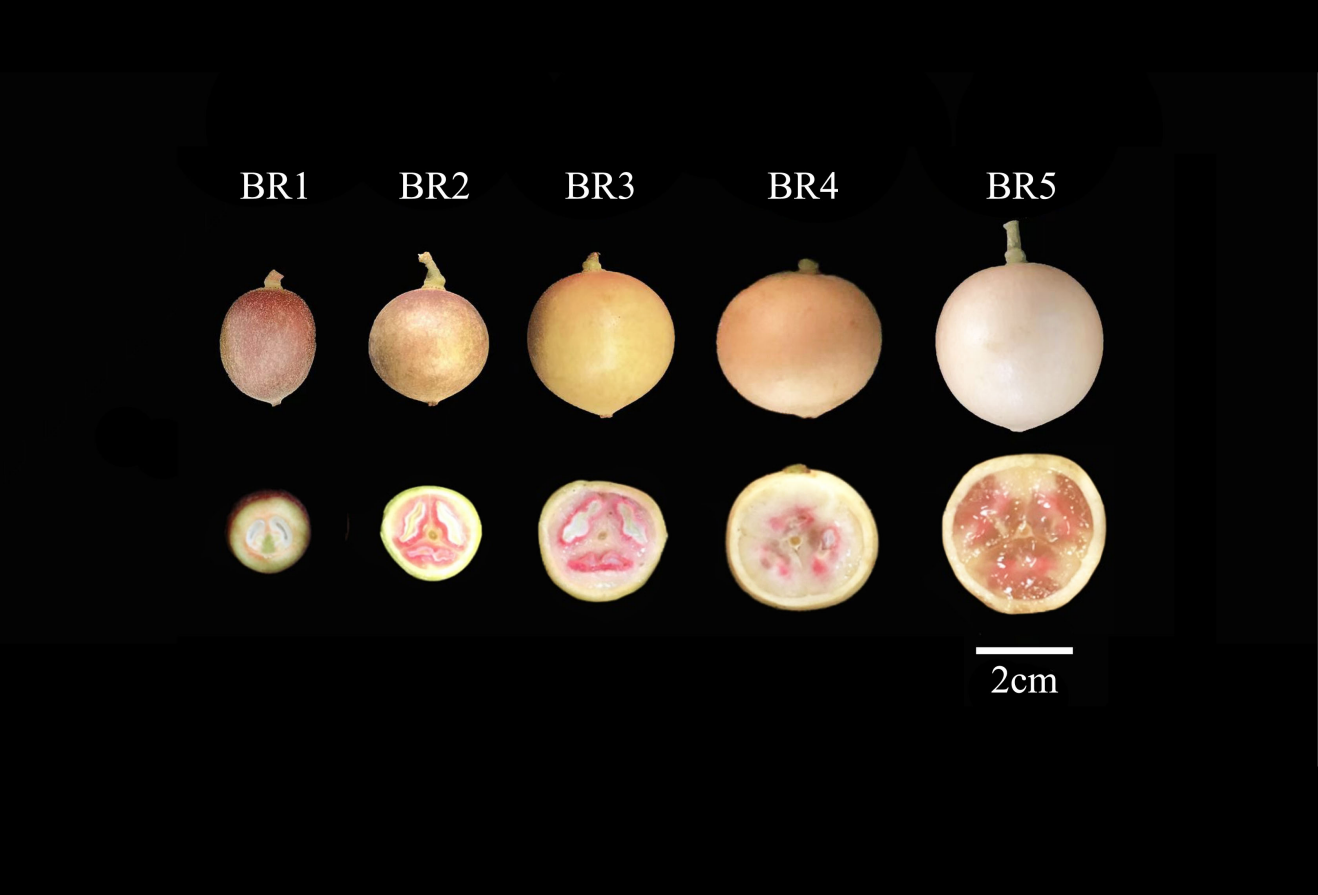
Five fruit profiles of the maturation process of BR *B. ramiflora*. From left to right, BR1 (30 days), BR2 (52 days), BR3 (73 days), BR4 (93 days), and BR5 (112 days) after full flowering, respectively. Scale bar = 2 cm.

BR (white pericarp and pink flesh) *B. ramiflora* fruits were collected from healthy fruit trees that were more than 10 years old and grown in the vicinity of Nasuo middle school in Fangchenggang, Guangxi, China (N 21°42′33″, E 108°6′29″, alt 20 m). The samples were collected at 30, 52, 73, 93, and 112 days after the flowering stage, respectively (**Figure 1**).

### 2.2 Sample preparation and metabolite extraction

We selected three trees with uniform growth and collected six fruits with the same maturity from each tree in five growing stages. Four pulps from the same period were randomly selected and mixed into one biological sample, and there were three biological replicates at each developmental stage. The samples were immediately stored in liquid nitrogen, brought back to the laboratory, and stored at −80°C, until the metabolites were extracted. Fifty milligrams of the sample was placed in a 1.5-mL centrifuge tube and added to 800 μL of extraction solution (water:methanol = 7:3, V:V, pre-cooled to −20°C), and 20 μL of the internal standard was added sequentially, ground in a tissue grinder (50 Hz, 5 min). The mixture was subjected to an ultrasonic water bath (4°C, 30 min), refrigerated (−20°C, 1 h), and centrifuged at 14,000 rpm for 15 min at 4°C using a low-temperature high-speed centrifuge (Centrifuge 5430, Eppendorf). The supernatant (600 μL) was taken and passed through a 0.22-μm filter membrane, and the filtrate sample was collected and placed in a sample bottle for ultrahigh-performance LC-MS/MS (UPLC-MS/MS) analysis (Chen et al., 2023).

### 2.3 UPLC-MS/MS analysis

Non-targeted metabonomics analysis identified the prepared extracts using a UPLC-HRMS/HRMS system (UPLC, Waters 2D UPLC, USA; HRMS, Thermo Fisher Scientific, USA), and data were collected in both positive and negative ion modes to improve the metabolite coverage (Di Guida et al., 2016). Five microliters of sample solutions was injected into a Hypersil GOLD aQ chromatographic column (100 mm * 2.1 mm, 1.9 μm, Thermo Fisher Scientific, USA). The UPLC mobile phase was composed of 0.1% formic acid aqueous solution (denoted as solvent A) and acetonitrile containing 0.1% formic acid (denoted as solvent B). The following gradient was used for elution: 0 min, 5% B; 2 min, 95% B; 22 min, 95% B; 27 min, 5% B solution. The overall process flow maintained a rate of 0.3 mL/min at a temperature of 40°C. The Q Exactive mass spectrometer was used for collecting primary and secondary mass spectrometry data. Parameters included the scanned mass-to-charge ratios (*m/z*) within the 150–1,500 range, a primary resolution of 70,000, an AGC of 1 × 10^6^, and a maximum injection time (MIT) set to 100 ms. The top three *m/z* peaks were selected based on the precursor ion intensity for fragmentation and secondary information collection. The secondary resolution was 35,000, with an AGC of 2 × 10^5^ and an MIT of 50 ms; the fragmentation energy (stepped NCE) was set to 20, 40, and 60 eV. The sheath gas and Aux gas flow rates of the ion source (ESI) were set to 40 mL/min and 10 mL/min, respectively. Spray voltage (|kV|) was set to 3.80 in the positive ion mode and 3.20 in the negative ion mode. The ion transfer tube temperature (capillary temperature) and the auxiliary gas heater temperature (aux gas heater temperature) were 320°C and 350°C, respectively.

### 2.4 Quality control measurement

The quality control (QC) sample was prepared by mixing equal volumes (20 μL) of each sample from five developmental stages, and the QC sample was measured using UPLC-MS/MS with the same parameter settings as the rest of the samples. The repeatability and stability of the UPLC-MS/MS analysis process were evaluated through the number of peaks, peak response intensity, chromatogram overlap, and principal component analysis (PCA) of QC.

### 2.5 MS data analysis

Data preprocessing was performed by importing the raw data collected from LC-MS/MS into Compound discoverer 3.1 (Thermo Fisher Scientific, USA). Data processing includes peak extraction, background peak labeling, intra-group and inter-group retention time correction, missing value filling, adduct ion merging, and metabolite identification, and then data on compound molecular weight, peak area, retention time, and identification results were exported in the end. The identification of metabolites was comprehensively referenced from multiple databases including BERRY Library, mzCloud, and ChemSpider [the Human Metabolome Database (HMDB), the Lipid Metabolites and Pathways Strategy, LipidMaps, and Kyoto Encyclopedia of Genes and Genomes (KEGG)].

The results obtained from Compound discoverer 3.1 were imported into metaX for data preprocessing, relative peak area was obtained using Probabilistic Quotient Normalization (PQN) (Di Guida et al., 2016), batch effect correction of the actual sample was performed via local polynomial regression fitting signal correction by QC-based robust LOESS signal correction (QC-RLSC) (Dunn et al., 2011) based on QC sample information, and the compounds of QC samples with a coefficient of variation (CV) greater than 30% were deleted.

### 2.6 MS statistical analysis

Statistical analyses of metabolites including PCA, hierarchical clustering analysis (HCA), fold change (FC), variable importance projection (VIP), metabolite classification, and functional annotations were performed using the metabonomic R software package metaX and the metabonomic information analysis process developed by Berry Hekang Gene Company (Wen et al., 2017). The Partial Least Squares Method Discriminant Analysis (PLS-DA) model was used to calculate the VIP of two principal components, which can measure the intensity of impact and explanatory power of different metabolite expression patterns on the classification discrimination of each sample group and assist in screening metabolic markers (Li et al., 2015). Log_2_ logarithmic conversion on data was performed, and the PLS-DA model between the comparative analysis group (two groups of samples) was established, with the scaling method of Par. Sevenfold cross-validation of the established PLS-DA model was performed, and 200 times response permutation testing was carried out to judge the model quality. Differential metabolites (DMs) were screened based on the VIP values of the first two principal components obtained from the PLS-DA model, combined with the results of FC and Student’s *t*-test obtained from univariate analysis. PCA and FC were converted by Log_2_, and the screening criteria were *p*-value <0.05, VIP ≥1, and FC ≥1.2 or ≤0.83. The increase or decrease of DMs was based on the five growing stages of BR *B. ramiflora*.

### 2.7 KEGG pathway enrichment analysis of metabolites

KEGG is a well-known and reliable database that explains molecular-level details of chemicals in organisms (Kanehisa et al., 2012) and was used to interpret the identified BR *B. ramiflora* metabolites. Annotated metabolites were mapped to the KEGG pathway database, and key pathways were identified by pathway enrichment analysis based on enrichment factors and the number of metabolites.

### 2.8 Statistical analysis

SPSS (22.0, IBM Corp., Armonk, NY, USA) software and OriginLab (2019, OriginLab Inc., Northampton, MA, USA) software were used for data statistical analysis and graphing, and expressed as the mean ± standard deviation (SD). Data were evaluated by one-way analysis of variance (ANOVA) using Tukey’s honestly significant difference (HSD) test (*p* < 0.05) (Feng et al., 2024).

## 3 Results and analysis

### 3.1 Statistical analysis of BR pulp metabolites

**Figure 2:**
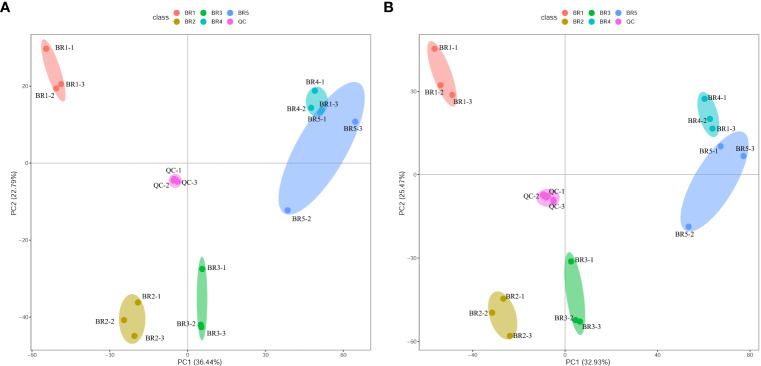
PCA of metabolites in the five developmental stages of BR *B. ramiflora.* QC represents a mixed equal amount of BR pulp samples. **(A)** Positive ion mode; **(B)** negative ion mode.

**Figure 3:**
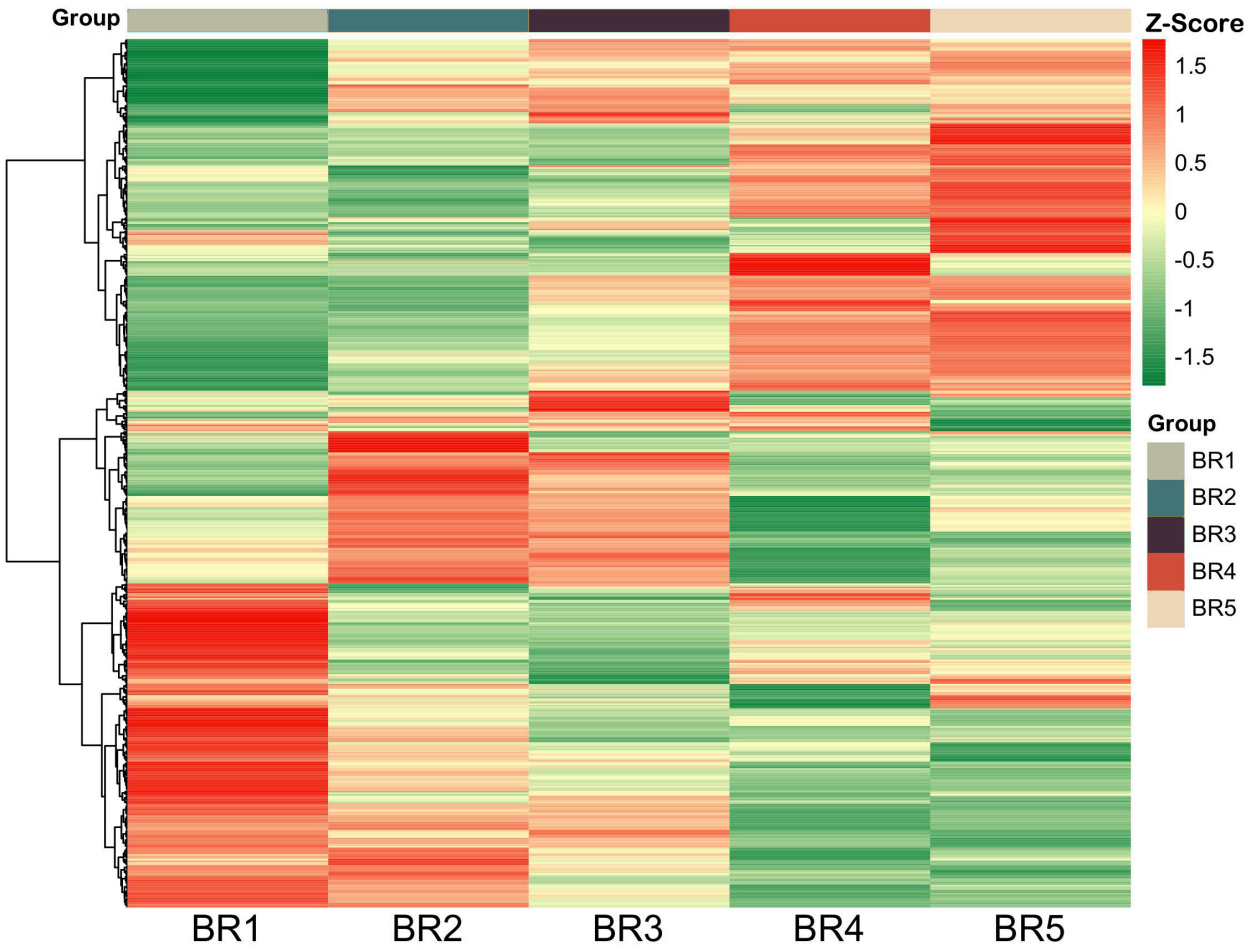
Hierarchical cluster analysis of metabolites identified from BR *B. ramiflora*. The color from green (low) to red (high) indicates the level of each metabolite. The *Z*-score represents the deviation from the mean by standard deviation units.

PCA showed a clear separation between samples of five developmental stages and QC, with significant differences (*p* < 0.05) as depicted in **Figure 2**. Among 18 samples, BR1 prominently separated from the rest, BR2 and BR3 were separated and proximal, while BR4 and BR5 were almost indistinguishable. The peak area of each metabolite was transformed by Log_2_, and subsequent HCA was performed to eliminate the influence of quantity on pattern recognition. HCA revealed five distinct groups related to BR1, BR2, BR3, BR4, and BR5, respectively (**Figure 3**). Overall, non-targeted metabolite analysis revealed the unique metabolite profiles of the five developmental stages of BR pulp, indicating the distinctive metabolic characteristics of the *B. ramiflora* fruit in different maturation stages.

### 3.2 Identification and classification of metabolites

A total of 536 metabolites in BR *B. ramiflora* were identified by non-targeted metabolite analysis utilizing LC-MS/MS, of which 361 were identified in positive ion mode and 201 were identified in negative ion mode, including 26 duplicates. The main substances affecting fruit flavor were 73 primary metabolites (12 carbohydrates, 3 organic acids, 7 amino acids and 8 derivatives, 2 vitamins, and 41 fatty acids) and 166 secondary metabolites (42 flavonoids, 8 phenols, 6 phenolic acids, 26 phenylpropanoids, 4 steroids and 5 derivatives, and 75 terpenoids).

### 3.3 Analysis of the dynamic changes in sugars and organic acids

Fruit taste mainly includes sweet, sour, and astringent, which is an important factor that consumers consider when buying fruits. These tastes, primarily determined by soluble sugars and organic acids, are intrinsically linked to fruit composition (Feng et al., 2024). Generally, sugar in fruits accumulates progressively from the unripe stage to full ripeness (Al-Maiman and Ahmad, 2002). BR fruits contain 12 kinds of carbohydrates, with the majority being L-sorbose, D-(+)-glucose, bis(methylbenzylidene) sorbitol, and sucrose (**Supplementary Figure 1**). Fructose was not detected, which may due to its low content. L-sorbose, which was the most abundant carbohydrate, showed a significant non-accumulation in the first three stages (30, 52, and 73 days), followed by a significant increase at 93 days (*p* = 0.033, FC = 2.327, VIP = 1.301), and reached the peak at 112 days. D-(+)-glucose was the second most abundant carbohydrate, and the accumulation trend was similar to L-sorbose. The contents of L-sorbose and D-(+)-glucose increased significantly at 93 days, indicating that the BR fruit entered the mature stage at 73 days and began to accumulate sugar rapidly. The content of bis(methylbenzylidene) sorbitol remained relatively steady, suggesting its negligible impact on the fruit’s texture or taste. The sucrose content was the smallest, showing an upward trend similar to L-sorbose and D-(+)-glucose in the first four developmental stages, further confirming that BR began to mature at 73 days. In addition, compared with BR4, the sucrose content of BR5 was decreased but was not significant, suggesting that sucrose was converted into glucose during the maturation period from 93 days to 112 days.

Citric acid was the main organic acid in the pulp of BR *B. ramiflora*, and malic and oxalic acids were undetectable. Citric acid initially increased and then decreased, which was consistent with the taste change of BR *B. ramiflora* pulp from astringent to sour in the early stage and from sour to sweet in the later stage. Citric acid decreased significantly in BR3 to BR4 (*p* = 0.007, FC = −2.974, VIP = 1.708), suggesting that BR began to mature at 73 days, which was consistent with the soluble sugars. Zhen (2015) considered that although the organic acid content could not directly determine the sweetness of fruits, the sugar–acid ratio significantly affects the flavor of fruits. As shown in **Table 1**, the soluble sugar [sucrose, L-sorbose, and D-(+)-glucose] content continuously increased from 61.67% in BR1 to 73.23% in BR5. Organic acids, mainly citric acid, increased slightly from BR1 to BR3, then significantly decreased from 21.897% in BR3 to 18.88% in BR4, and finally showing no significant increase in BR4 and BR5. With the change in the content of soluble sugar and organic acid, the sugar–acid ratio continually increased from 2.91 in BR1 to 3.87 in BR4, subsequently sliding to 3.78 in BR5. These results indicated that the change of sugar–acid ratio affected the taste of the BR *B. ramiflora* fruit, and the main reason was the increase of soluble sugar.

**Table 1:** Sugars, acids, and basic taste qualities of BR pulp at five mature stages.

Growing stage	Sugars				Acids		Sugar–acid ratio
	Soluble sugar (%)	Sucrose	L-sorbose	D-(+)-glucose	Titratable acid (%)	Citric acid	
BR1	61.66 ± 0.12c	18.65 ± 0.51c	22.58 ± 0.18c	20.42 ± 0.49d	21.17 ± 0.25a	21.17 ± 0.25a	2.91 ± 0.04b
BR2	63.90 ± 1.52bc	20.28 ± 0.77bc	22.56 ± 0.29c	21.06 ± 0.56cd	21.80 ± 0.14a	21.80 ± 0.14a	2.93 ± 0.09b
BR3	65.70 ± 1.93b	21.48 ± 0.57ab	22.61 ± 0.95c	21.61 ± 0.48c	21.90 ± 0.03a	21.90 ± 0.03a	3.00 ± 0.09b
BR4	72.94 ± 1.20a	24.15 ± 0.70a	25.08 ± 0.36b	23.71 ± 0.15b	18.88 ± 0.44b	18.88 ± 0.44b	3.86 ± 0.05a
BR5	73.23 ± 3.70a	22.31 ± 3.02ab	26.22 ± 0.62a	24.69 ± 0.33a	19.38 ± 1.31b	19.38 ± 1.31b	3.78 ± 0.18a

Sugar–acid ratio is soluble sugar divided by titratable acid. Different letters on the number meant significant differences between growing stages (p < 0.05).

### 3.4 Analysis of the dynamic changes in amino acids

Amino acids, sugars, and organic acids are the primary metabolic products of fruits that determine fruit quality (Zhang et al., 2010). The composition and content of amino acids are important indexes to evaluate nutritional quality and taste. In this study, L-phenylalanine was the main amino acid affecting flavor, followed by L-tyrosine and DL-arginine in lower proportions (**Supplementary Figure 2**). L-phenylalanine decreased slightly in the first four developmental stages and increased significantly only in the last developmental stage, which may improve the taste of fruit; DL-arginine did not change significantly in the first three development stages, and then increased significantly, which indicated that DL-arginine had a certain effect on pulp quality. There was no significant difference in L-tyrosine among the five developmental stages, but it increased at 112 days. The contents of all three amino acids increased at the final developmental stage, indicating that it may be a reliable indicator of maturation. The results showed that the accumulation of amino acids increased during the mature stage, which played an important role in the fruit taste.

### 3.5 Analysis of the dynamic changes in fatty acids

Fatty acids are essential components that contribute to fruit quality (Zishun et al., 2016). A total of 38 fatty acids were identified in the BR pulp, mainly oleamide, A-eleostearic acid, and corchorifatty acid F, and their content variations are shown in **Supplementary Figure 3**. The oleamide content remained stable in the first three growth stages and gradually increased from 73 days to 112 days. A-eleostearic acid did not show significant fluctuations in five developmental stages. Corchorifatty acid F showed a downward trend, with significant reductions in BR2 vs. BR1 (*p* = 0.0007, FC = −2.887, VIP = 1.347) and BR5 vs. BR4 (*p* = 0.1293, FC = 1.407, VIP = 1.7769). The increase of oleamide in the late stage, as well as the significant decrease of corchorifatty acid F, played an important role in improving the taste of BR *B. ramiflora*.

### 3.6 Analysis of the dynamic changes in flavonoids

Flesh color is usually determined by carotenoids, anthocyanins, and flavonoids, which are also pigments of various plants (Ferrer et al., 2008). The synthesis of anthocyanin is related to flavonoids and phenylpropanoids, which are precursors of anthocyanin synthesis. Flavonoids provide protection for plants under diverse stress conditions and provide beneficial health effects in humans (Jaakola, 2013; Zhang et al., 2014). The biosynthetic pathway of flavonoids plays a pivotal role in the production and regulation of anthocyanins, proanthocyanidins, and flavonols (Wang et al., 2019).

A total of 42 flavonoids were identified in BR flesh, with the absence of carotenoids. Rhusflavanone, procyanidin B1, and catechin constituted the primary flavonoids. Rhusflavanone, the most abundant one in content, progressively increased during the whole fruit maturation. Rhusflavanone underwent a significant increase across different developmental stages, BR3 vs. BR2 (*p* = 0.0002, FC = 1.926, VIP = 1.234) and BR4 vs. BR3 (*p* = 0.0004, FC = 2.778, VIP = 1.230), indicating that rhusflavanone was evident from 73 days to 112 days and culminated in elevated quantities in the mature stage. Procyanidin B1 showed a decreasing trend, suggesting that the reduction of bitterness in the pulp was related to its gradual reduction. Catechin, a preliminary substance for procyanidin B1 synthesis, displayed a decreasing trend, which corresponded to the reduced synthesis of procyanidin B1. The gradual decrease in procyanidin B1 and catechins may be related to the reduction of anthocyanidin synthesis or upstream substrate synthesis.

Ten metabolites related to the biosynthetic processes of flavonoids, including anthocyanins, were found: naringenin chalcone, naringenin, eriodictyol, dihydroquercetin, kaempferol, quercetin, (+)-catechin hydrate, (−)-catechin gallate, procyanidin B1, and rhusflavanone. Ten metabolites showed an overall downward trend during development (**Supplementary Figure 4**), with the accumulation of the contents of each substance mainly in the first two periods, indicating that anthocyanins were mainly synthesized in the earlier period. The content of dihydroquercetin, an essential precursor for anthocyanin synthesis, was significantly decreased in BR5 vs. BR4 (*p* = 0.020, FC = −1.338, VIP = 1.209), speculating that the increase of its content was an important reason for the pink flesh of the BR fruit. Kaempferol experienced a significant decrease in BR5 vs. BR4 (*p* = 0.021, FC = −1.339, VIP = 1.256), indicating a diminished synthesis of anthocyanins. Thus, anthocyanin synthesis gradually decreased in the late stage.

### 3.7 Analysis of the dynamic changes in polyphenolics

Polyphenolics can affect the color and biological characteristics of fruits, which is an important index to evaluate fruit quality. Generally, unripe fruits are unpalatable due to the presence of polyphenolics, resulting in a bitter and astringent taste. The reduction of polyphenolic content during fruit ripening will lessen these bad flavors and lead to a better fruit flavor (Aldhanhani et al., 2022). Therefore, the maturity and harvest time of fruits can be judged by the content of polyphenolics (Cuthbertson et al., 2012; Prakash et al., 2020). Seven high-content polyphenolic metabolites were identified in the BR pulp, 2-hydroxycinnamic acid, caffeic acid, phloretin, hexylcinnamaldehyde, dihydromethysticin, demethoxyyangonin, and apocynin. Polyphenolic content gradually decreased as the fruit ripened (**Supplementary Figure 5**). A significant decrease appeared at the stage from BR4 to BR3, and a higher polyphenolic content from BR1 to BR3 than from BR4 and BR5 indicated that the synthesis and accumulation of polyphenolic substances mainly happen in the early stages of BR *B. ramiflora* development.

### 3.8 Analysis of the dynamic changes in vitamins

Two vitamin metabolites, pantothenic acid and DL-thioctic acid, were identified (**Supplementary Figure 6**). Pantothenic acid is a precursor of coenzyme A synthesis and widely distributed in plants (Serrano-García et al., 2022). The pantothenic acid content remained relatively stable in 30, 52, 73, and 93 days, and remarkably increased in the last stage (*p* = 1.8453, FC = 1.043, VIP = 1.557). In DL-thioctic acid, there was no significant difference in 30, 52, and 73 days, but there was a significant decrease in BR4 vs. BR3 (*p* = 2.3727, FC = −3.359, VIP = 1.644). The results suggested that pantothenic acid and DL-thioctic acid may affect the taste of the BR pulp in the later maturation stages.

### 3.9 Differential metabolites at five different maturity stages

**Figure 4:**
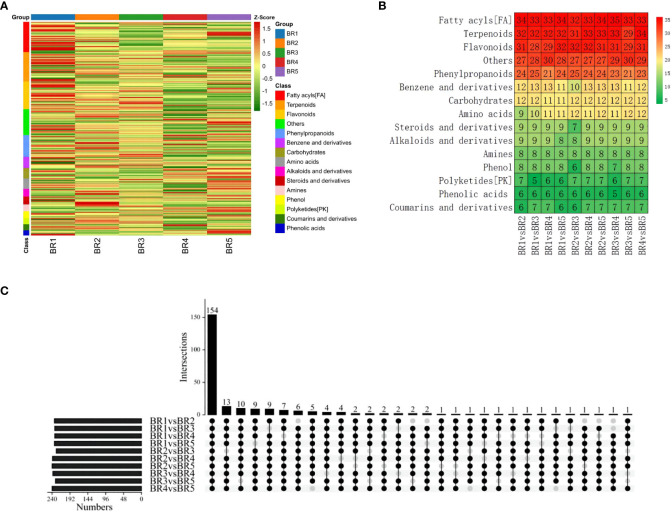
Identification and classification of metabolic discrepancy. **(A)** Hierarchical cluster analysis of metabolites identified from BR *B. ramiflora*. The color from green (low) to red (high) indicates the level of each metabolite. The *Z*-score represents the deviation from the mean by standard deviation units. **(B)** Number of differential metabolites. **(C)** A total of 154 common differential accumulated metabolites by the UpSet plot.

To identify DMs in five growing stages of BR *B. ramiflora*, we selected metabolites based on *p*-value < 0.05, VIP ≥1, Log_2_FC ≥1.2, and Log_2_FC ≤0.83. In total, 247 DMs were identified, which could be divided into 15 classes (**Figure 4A**). The metabolites of 10 groups were compared and analyzed, and major DMs were mainly fatty acyls (FA), terpenoids, flavonoids, phenylpropanoids, and others (**Figure 4B**).

A total of 154 common DMs were identified from 10 groups in five stages by the UpSet plot analysis (**Figure 4C**), namely, 21 FAs, 21 flavonoids, 20 terpenoids, 14 phenylpropanoids, 10 carbohydrates, 8 amines, 7 alkaloids and derivatives, 7 benzene and derivatives, 7 steroids and derivatives, 6 amino acids, 5 phenols, 4 phenolic acids, 4 coumarins and derivatives, 3 polyketides, and 17 others. The comparison analysis of metabolites at different maturity stages showed 234, 233, 233, and 234 DMs in BR1 vs. BR2, BR3, BR4, BR5; 229, 239, and 240 DMs in BR2 vs. BR3, BR4, and BR5; 238 and 231 DMs in BR3 vs. BR4 and BR5; and 240 DMs in BR4 vs. BR5.

Flavonoids and terpenoids affect fruit coloration, and FAs played an important role in fruit development and affected the structure and function of plant cell membrane (Zheng et al., 2022). The significant variation of flavonoids, FAs, and terpenoids may explain fruit coloring and the morphological alterations in fruit cross-sections and provide important support for fruit development. Sugars, organic acids, and amino acids were the major compounds contributing to sensory variation. Five carbohydrates exhibited a continuous increase, namely, D-(+)-glucose, lactose, L-sorbose, lusitanicoside, and uridine 5’-diphosphogalactose, indicating that carbohydrate synthesis was active during the maturation process of BR *B. ramiflora*. Additionally, five amino acids were actively synthesized (zeatin-7-n-glucoside biocytin, L-alanyl-l-proline, DL-arginine, L-phenylalanine, and L-tyrosine) and one amino acid was degraded (methionine sulfoxide) during fruit ripening.

### 3.10 KEGG enrichment analysis of DMs

The comprehensive analysis of metabolites in five mature stages of BR *B. ramiflora* was performed using the KEGG database, and the main metabolic pathways were elucidated (**Supplementary Figure 7**). There were 37 metabolic pathways, mainly sugar and acid and amino acid metabolism. Sugar and acid metabolism included glycolysis/gluconeogenesis, fructose and mannose metabolism, pyruvate metabolism, and citrate cycle, and the metabolites involved in the glycometabolism pathway were mainly L-sorbose, citric acid, D-(−)-salicin, and 2-isopropylmalic acid. Twelve pathways were established in amino acid metabolism, namely, 2-oxocarboxylic acid metabolism; alanine, aspartate, and glutamate metabolism; beta-alanine metabolism; biosynthesis of amino acids; cyanoamino acid metabolism; glycine, serine, and threonine metabolism; phenylalanine metabolism; phenylalanine, tyrosine, and tryptophan biosynthesis; tryptophan metabolism; tyrosine metabolism; valine, leucine, and isoleucine biosynthesis; and aminoacyl-tRNA biosynthesis. Twelve metabolic pathways of amino acid synthesis, such as amino acid biosynthesis and L-phenylalanine, tyrosine, and tryptophan biosynthesis, share common metabolic pathways. A total of nine metabolites were identified, and L-tyrosine and L-tryptophan were the most active metabolites involved in six amino acid metabolic pathways. KEGGs found that DMs were mainly enriched in plant hormone signal transduction, linoleic acid metabolism, oxidative phosphorylation, phenylalanine, tyrosine and tryptophan biosynthesis, and citrate cycle. Metabolite analysis showed that many metabolites were involved in the synthesis of amino acids, which may significantly improve the nutritional value and taste of the BR fruit during ripening.

**Figure 5:**
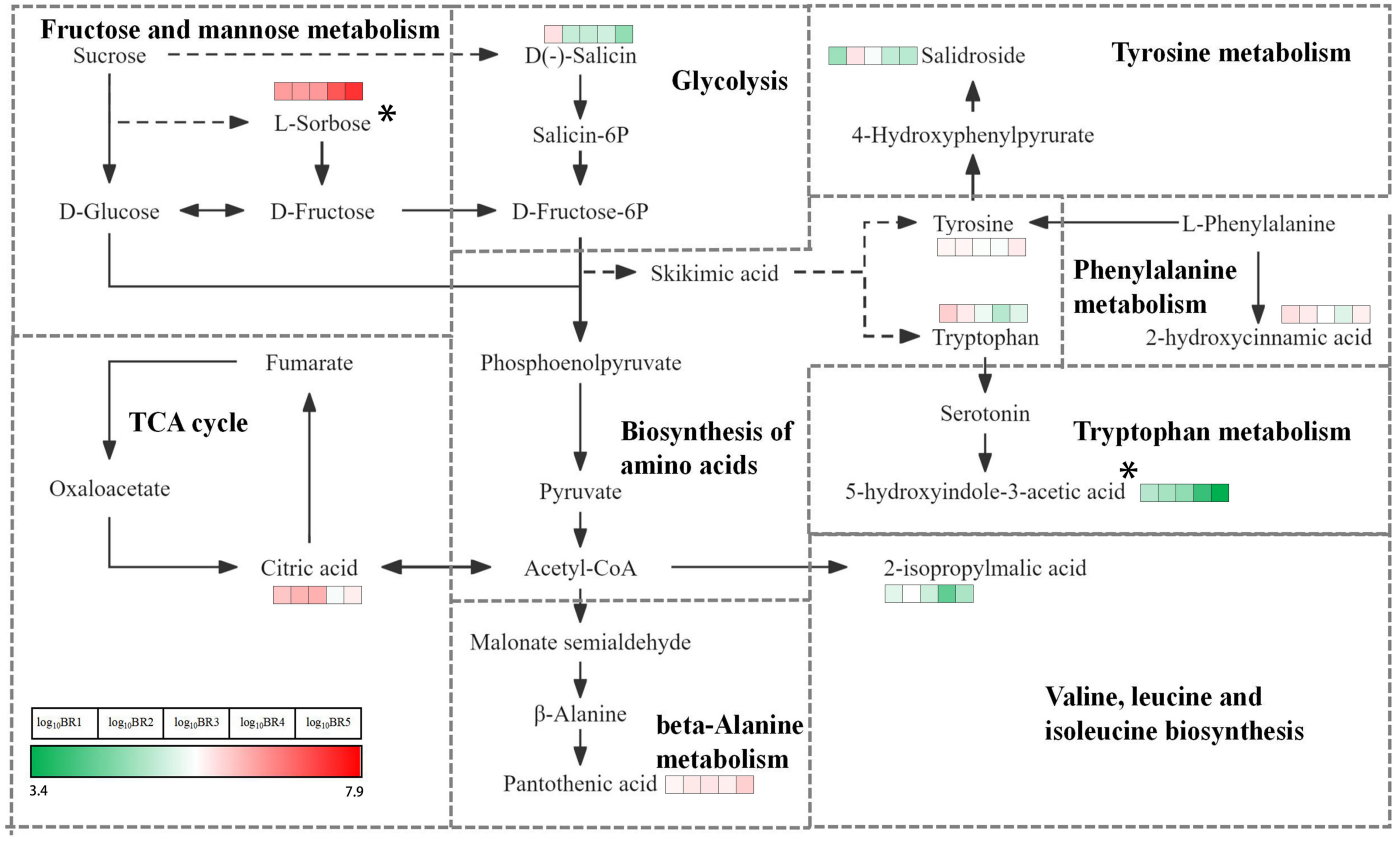
KEGG map of key DMs in BR *B. ramiflora*. This map was constructed based on the KEGG pathway and literary references. Colored boxes in front of each metabolite indicate log_10_BR1, log_10_BR2, log_10_BR3, log_10_BR4, and log_10_BR5 values according to the color scale. * represents the taste biomarker.

It provided a comprehensive analysis of the KEGG pathway based on carbohydrate metabolism and amino acid biosynthesis pathway, and explained the mechanism of flavor change of the BR fruit (**Figure 5**). L-sorbose, a significant sugar in BR, was active in the fructose and mannose metabolism pathway with a significant accumulation from BR1 to BR5, showing that L-sorbose played a key role in the ripening and flavor change of the BR fruit. Conversely, D-(−)-salicin, involved in the glycolysis pathway, continuously decreases from BR1 to BR5, suggesting an ongoing breakdown during the whole maturation of the BR fruit. 2-Isopropylmalic acid entered the valine, leucine, and isoleucine biosynthesis pathway via phosphoenolpyruvate, and undergoes a notable change throughout the maturation, showing a trend of first increasing, then decreasing, and finally increasing, representing high synthesis and decomposition activity. Two dynamic amino acids, L-tryptophan and L-tyrosine, were simultaneously found in the amino acid biosynthesis pathway. L-tyrosine remained consistent throughout the five maturation stages, while L-tryptophan showed a significant decrease from BR1 to BR4 and a significant increase from BR4 to BR5, suggesting that L-tryptophan continuously decomposes and synthesizes during BR fruit maturation. 5-Hydroxyindole-3-acetic acid, a metabolite found in the tryptophan metabolism pathway, continuously and significantly decreased from BR1 to BR5, suggesting that 5-hydroxyindole-3-acetic acid potentially played a major role in the flavor changes of BR fruit ripening. 2-Hydroxycinnamic acid was identified, entering the phenylalanine metabolism pathway via L-tyrosine, with a significant reduction in content from BR1 to BR4 and an increase from BR4 to BR5. Salidroside was found to enter the tryptophan metabolism pathway via L-tyrosine, decreasing significantly from BR1 to BR2 and decreasing continuously from BR2 to BR5. The changes in salidroside and 2-hydroxyquinamic acid indicated their dynamic role in synthesis and decomposition during the ripening process of BR fruits. In addition, citric acid and pantothenic acid, which respectively entered the citrate cycle pathway through acetyl-CoA and the beta-alanine metabolism pathway, were involved in the synthesis and decomposition of metabolites during the ripening of BR *B. ramiflora*.

### 3.11 Potential taste biomarker

**Figure 6:**
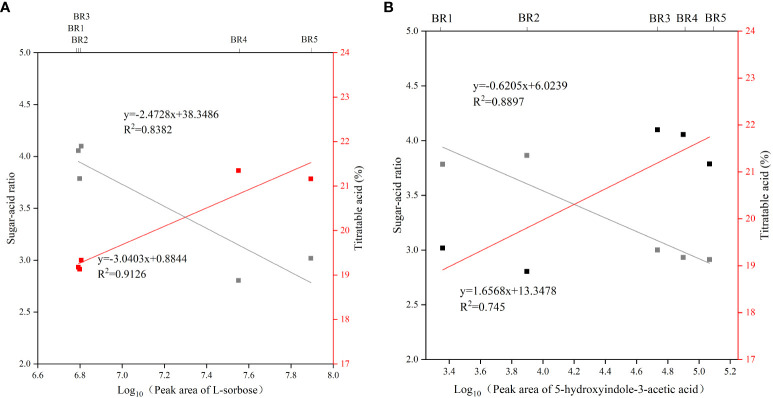
Correlation between potential taste biomarker, sugar–acid ratio, and titratable acids in BR *B. ramiflora.* **(A)** L-sorbose; **(B)** 5-hydroxyindole-3-acetic acid.

Of the 154 common metabolites, only L-sorbose consistently increased with increasing BR maturity, resulting in a persistent increase in the sugar–acid ratio, presumably suggesting that L-sorbose may be a taste biomarker for BR fruits. Interestingly, KEGG enrichment analysis found that 5-hydroxyindole-3-acetic acid in the tryptophan metabolism pathway consistently decreased with increasing BR maturity. Further analysis showed that L-sorbose was positively correlated with sugar–acid ratio (*R* ^2^ = 0.9126) and negatively correlated with titratable acid (*R* ^2^ = 0.8382), and 5-hydroxyindole-3-acetic acid was positively correlated with sugar–acid ratio (*R* ^2^ = 0.8897) and negatively correlated with titratable acid (*R* ^2^ = 0.745) (**Figure 6**).

Carbohydrate is the main energy source for plant growth and development, and L-sorbose was the main carbohydrate of the BR fruit (Dhungana and Braun, 2021). 5-Hydroxyindole-3-acetic acid is a derivative of tryptophan and is a precursor to many natural products, such as pigments, alkaloids, hormones, and cell wall components in plants (Maeda and Dudareva, 2012). In conclusion, these results suggested that L-sorbose and 5-hydroxyindole-3-acetic acid may be key taste biomarkers of the BR fruit.

## 4 Discussion

*B. ramiflora*, mostly found in tropical forests with wild distribution, has not yet been commercially planted and utilized (Ramjan and Raghavan, 2018). There were significant differences between the flowering and fruiting stages from south to north. Generally, *B. ramiflora* bloom and bear fruit approximately 45 days earlier in Southeast Asian regions than those in mainland China (Huang et al., 2024). The fruits ripen between late May and early August, similar to litchi and longan. It has the potential to alleviate fruit shortages of this season, given the different breeding and fruiting periods of different provinces (Chen et al., 2023). Understanding the phenological period of *B. ramiflora* is not only valuable to their harvest time, but also beneficial to their commercial cultivation (Fyfe et al., 2023). Through the non-targeted metabolomics analysis of five typical growth stages of BR *B. ramiflora*, we have identified key metabolic changes that characterize each phase of development. The results showed that L-sorbose and D-(+)-glucose increased significantly at 93 days, indicating that the BR fruit entered the mature stage at 73 days and began to accumulate sugar rapidly. Citric acid displayed a trend of initial increase and then decrease, which was consistent with the taste change of the BR *B. ramiflora* pulp from astringency to sour in the early stage and from sour to sweet in the later stage. By analyzing the contents of sugars, organic acids, amino acids, fatty acids, flavonoids, polyphenolics, and vitamins in different fruit stages, we found that BR3 to BR4 may be the key period of respiratory climacteric. This is consistent with previous studies; maturity III (98 days after flowering) has good quality and high storage resistance, which can better meet the fresh marketing and storage of the *B. ramiflora* fruit after harvest (Kong et al., 2024).

Harvest maturity is an important factor affecting fruit quality and storability (Khalil et al., 2023). Understanding the mechanism of fruit ripening is critical for fruit storage and quality improvement (Zhang et al., 2021; Wang et al., 2022). In this study, non-target metabolome analysis was used to clarify the metabolic change of the BR *B. ramiflora* fruit (peel white, pulp pink) during five ripening stages and to reveal the key substances of taste change. A total of 536 metabolites were identified. The main primary metabolites were L-sorbose, D-(+)-glucose, citric acid, L-phenylalanine, oleamide, and α-eleostearic acid; the secondary metabolites were mainly rhusflavanone, procyanidin B1, caffeic acid, and pantothenic acid. The discovery of key biomarkers is important for the early prediction, diagnosis, and classification of fruit quality. Deng et al. (2023) explained the main reasons for fruit taste changes by mining key biomarkers of rambutan. L-sorbose and 5-hydroxyindole-3-acetic acid were closely associated with sugar–acid ratio and titratable acids in fruit development, demonstrating that they may be key taste biomarkers of the BR fruit. Our study provides not only a new insight into the metabolic changes during *B. ramiflora* ripening but also an important reference for the improvement of *B. ramiflora* quality.

*B. ramiflora* has high medicinal and health value (Inta et al., 2013). However, the use of natural plant products for medicinal purposes is still challenging because of their low content and complex structure and issues in mass production and synthesis (Sirirungruang et al., 2022). Fortunately, advances in technology related to synthetic chemistry, pharmaceutical chemistry, pharmacology, and molecular biology have made it much easier to discover new natural plant products (Li et al., 2023). In this study, 536 metabolites in BR *B. ramiflora* were identified by non-targeted metabolite analysis utilizing LC-MS/MS. According to different medicinal needs, we can determine the optimal fruit picking time by non-targeted metabolomes (Fang et al., 2023). The discovery of metabolites will provide a useful reference to further develop the medicinal value of *B. ramiflora*.

## 5 Conclusions

In conclusion, different from the previous physiological and biochemical studies on various stages of fruit ripening, this study provided a new perspective for understanding the critical metabolites during the various stages of *B. ramiflora* ripening. This study showed that the changes of metabolites at different maturity stages were closely associated with *B. ramiflora* fruit quality. As a result, through the non-targeted metabolomics analysis of five typical growth stages of the BR *B. ramiflora* fruit, 536 metabolites and 154 common metabolites were screened out. On the basis of pathway enrichment analysis, its characteristic metabolites and potential flavor biomarkers were identified, and its possible mechanisms were explained. The results provided new metabolic lines of evidence for taste variation during the ripening process of *B. ramiflora*. Future research can be combined with genomics and transcriptomics to better understand the relationship between gene expression, protein synthesis, and metabolite changes, so as to further explore the molecular basis of *B. ramiflora* fruit quality formation.

## Data availability statement

The original contributions presented in the study are included in the article/**Supplementary Material**. Further inquiries can be directed to the corresponding authors.

## Author contributions

CY: Data curation, Formal analysis, Methodology, Project administration, Resources, Writing – original draft. JQC: Data curation, Formal analysis, Methodology, Project administration, Resources, Writing – original draft. YZ: Supervision, Validation, Writing – review & editing. JH: Investigation, Resources, Validation, Writing – original draft. HW: Data curation, Formal analysis, Software, Validation, Writing – review & editing. JC: Conceptualization, Formal analysis, Funding acquisition, Investigation, Methodology, Project administration, Resources, Supervision, Validation, Visualization, Writing – review & editing.
