# Supplementary material for: Non-targeted metabolomics reveals the taste variations during Baccaurea ramiflora Lour. fruit maturation
Source: Front Plant Sci. 2024 Jul 8;15:1420231. doi: 10.3389/fpls.2024.1420231 (PMC11260711; doi:10.3389/fpls.2024.1420231)
Supplement: Supplementary file 1 [file Image_1.pdf]

Supplement figure

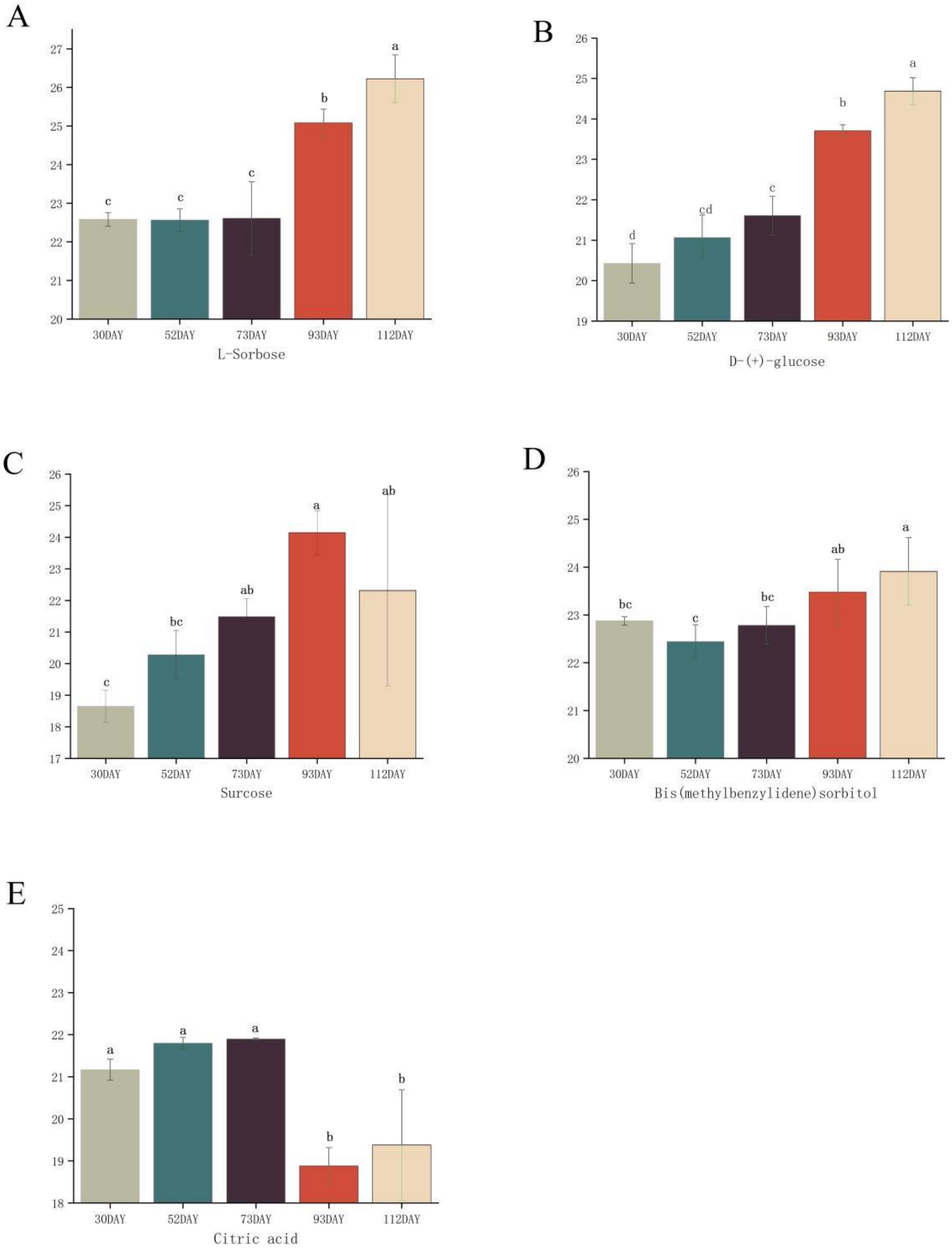

Fig.S1. Soluble sugar and Citric acid content of five different ripening stages.

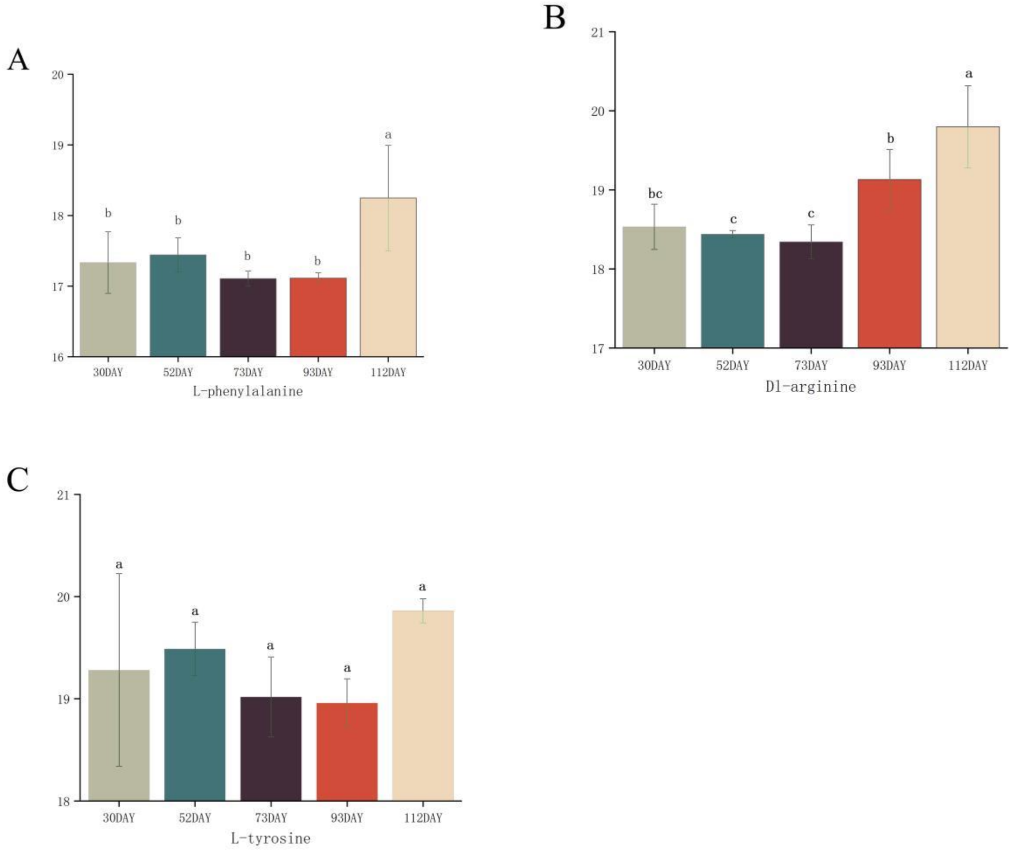

Fig. S2 Amino acids and their derivatives content of five different ripening stages.

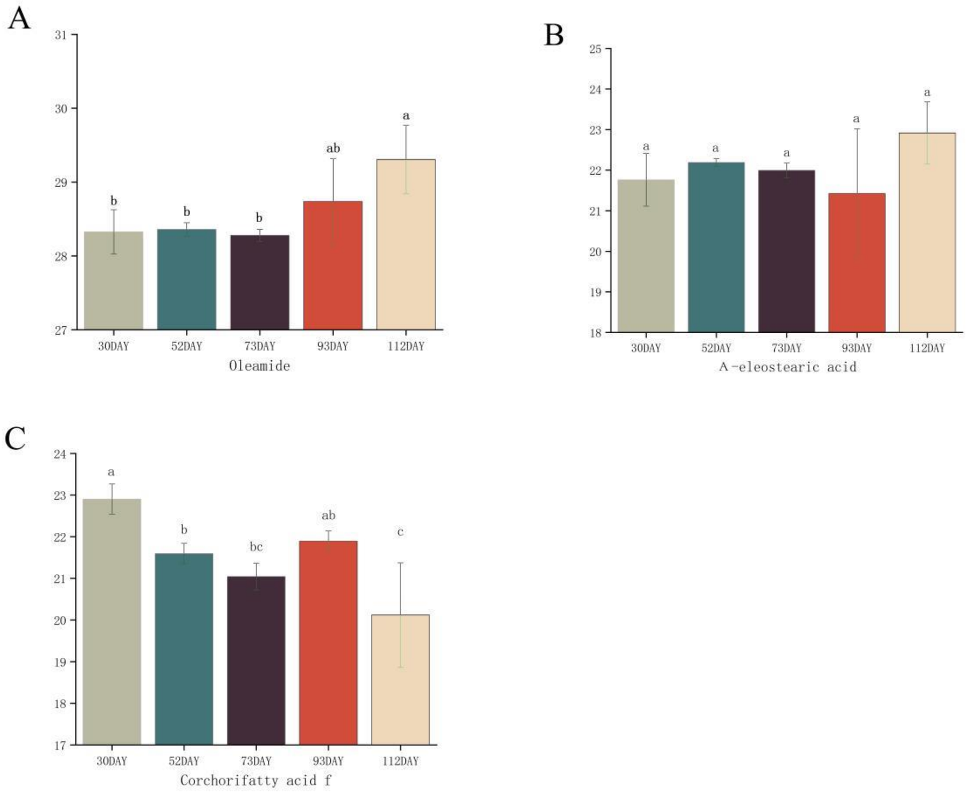

Fig.S3. Fatty acids content of five different ripening stages .

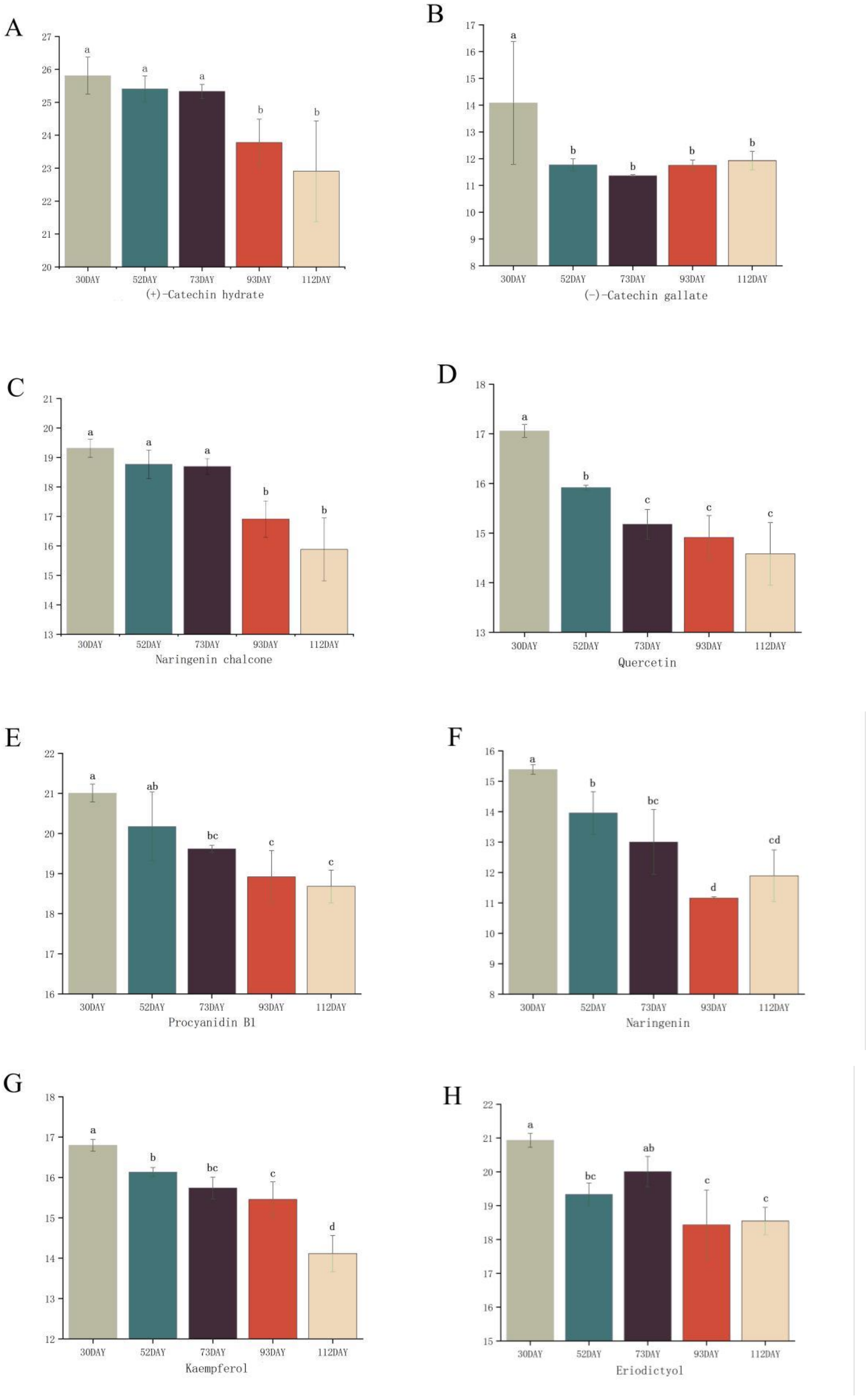
A
(+)-Catechin hydrate
B
(-)-Catechin gallate
C
Naringenin chalcone
D
Quercetin
E
Procyanidin B1
F
Naringenin
G
Kaempferol
H
Eriodictyol
30DAY
52DAY
73DAY
93DAY
112DAY

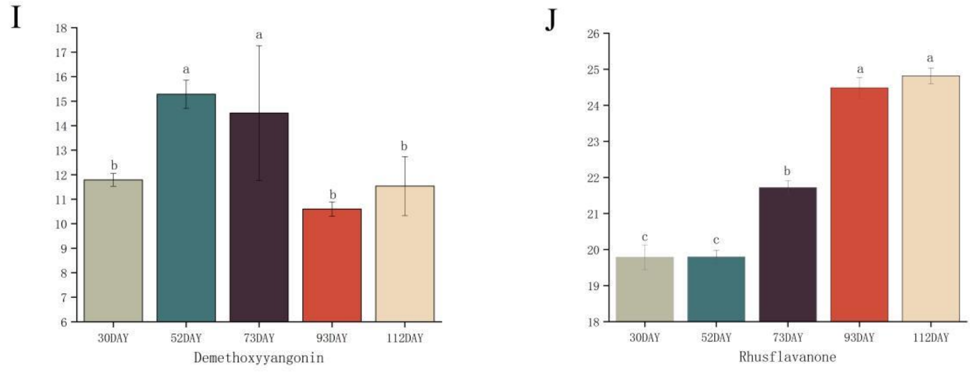

Fig.S4. Flavonoids content of five different ripening stages.

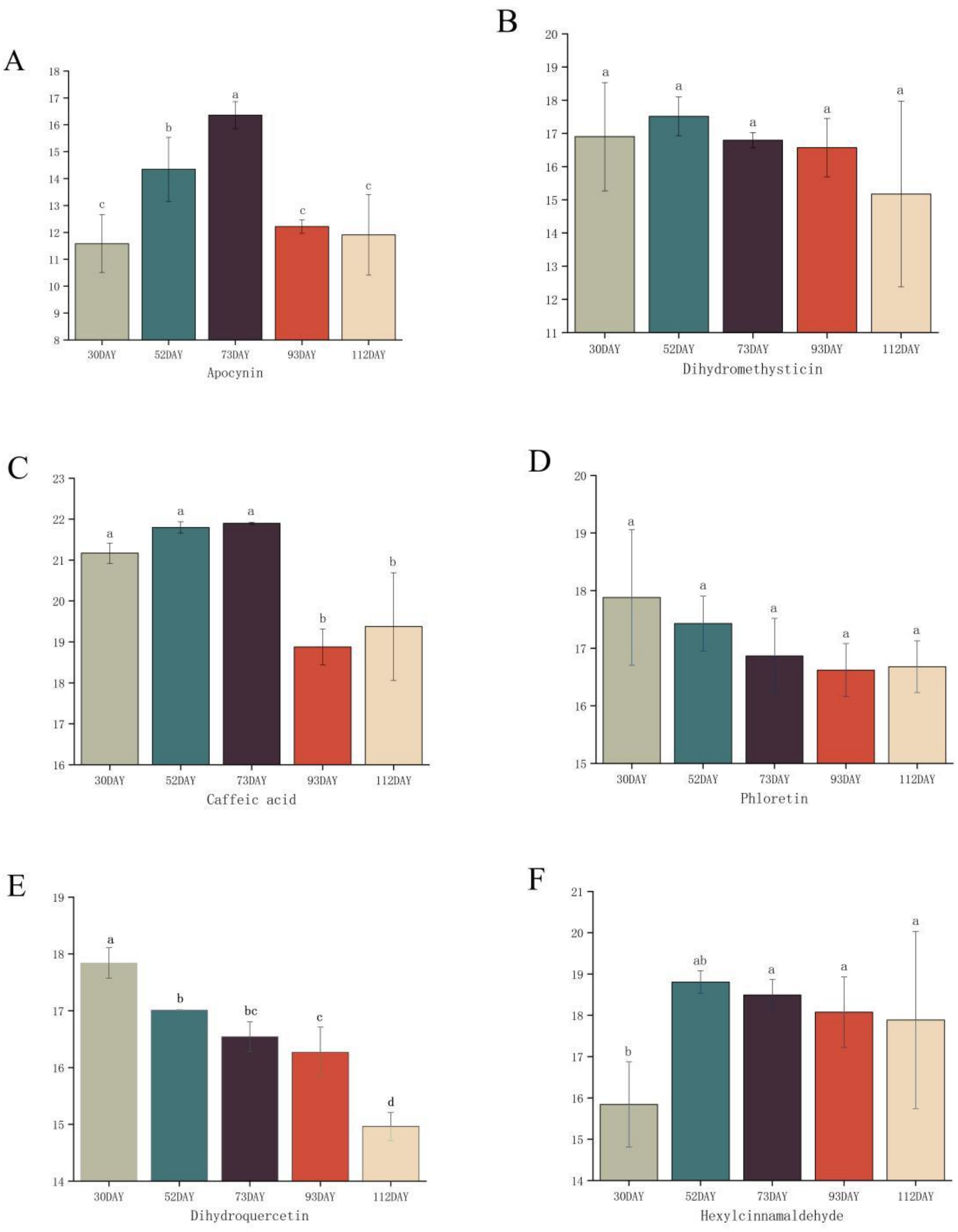

G

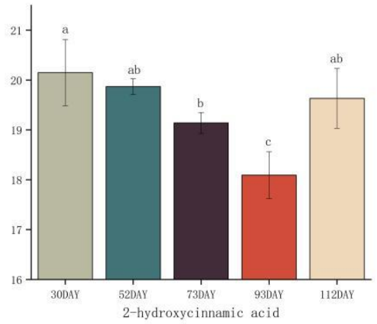

Fig.S5. Polyphenolics content of five different ripening stages.

A

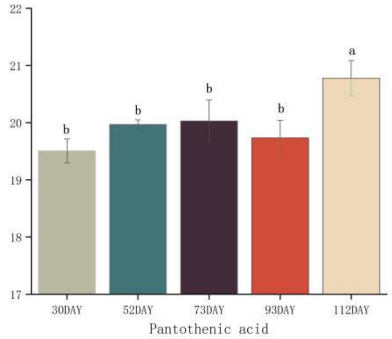

B

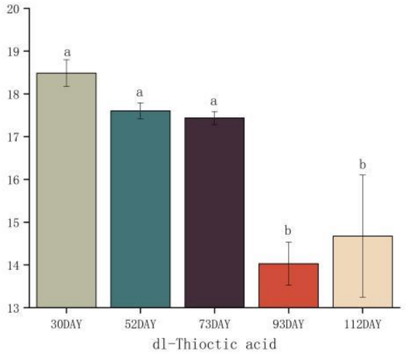

Fig.S6. Vitamins content of five different ripening stages.

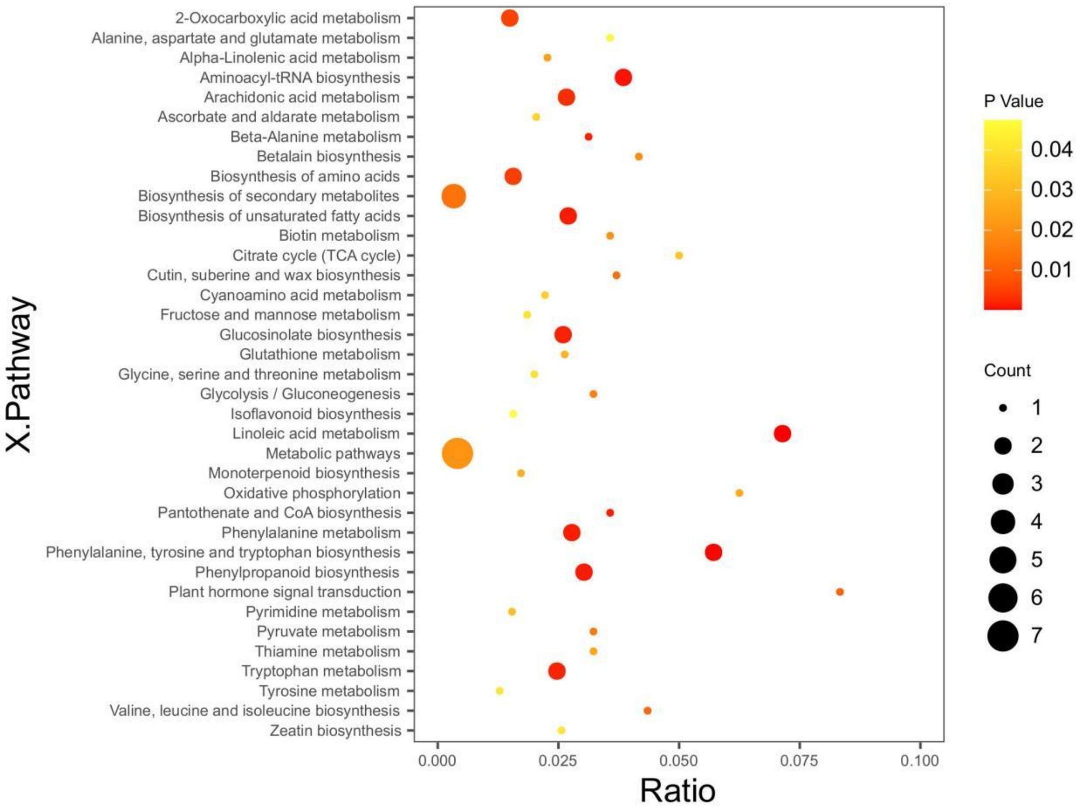

Fig.S7. The main metabolic pathways.
